# TOPII and chromosome movement help remove interlocks between entangled chromosomes during meiosis

**DOI:** 10.1083/jcb.201803019

**Published:** 2018-12-03

**Authors:** Marina Martinez-Garcia, Veit Schubert, Kim Osman, Alice Darbyshire, Eugenio Sanchez-Moran, F. Chris H. Franklin

**Affiliations:** 1 School of Biosciences, University of Birmingham, Birmingham, UK; 2 Leibniz Institute of Plant Genetics and Crop Plant Research, Gatersleben, Germany

## Abstract

During the zygotene stage of meiosis, normal progression of chromosome synapsis and homologous recombination frequently lead to the formation of structural interlocks between entangled chromosomes. The persistence of interlocks through to the first meiotic division can jeopardize normal synapsis and occasionally chromosome segregation. However, they are generally removed by pachytene. It has been postulated that interlock removal requires one or more active processes, possibly involving topoisomerase II (TOPII) and/or chromosome movement. However, experimental evidence has been lacking. Analysis of a hypomorphic *topII* mutant and a meiosis-specific *topII* RNAi knockdown of *Arabidopsis thaliana* using immunocytochemistry and structured illumination microscopy (SIM) has now enabled us to demonstrate a role for TOPII in interlock resolution. Furthermore, analysis using a nucleoporin *nup136* mutant, which affects chromosome movement, reveals that although TOPII activity is required for the removal of some interlock structures, for others, chromosome movement is also necessary. Thus, our study demonstrates that at least two mechanisms are required to ensure interlock removal.

## Abstract (precis)

During meiosis, unrelated chromosomes frequently become interlocked, and these structures must be removed for complete synapsis and normal chromosome segregation. Martinez-Garcia et al. show that the active removal of interlocks requires topoisomerase II and chromosome movement.

## Introduction

In sexual reproduction, gamete formation is dependent on meiosis, a specialized cell division pathway involving a single round of DNA replication followed by two rounds of division. At the onset of meiotic prophase I, pairs of newly replicated sister chromatids of each homologue become organized into linear looped arrays conjoined at the loop bases by a protein axis. Homologue pairs then align and undergo synapsis through the formation of the synaptonemal complex (SC), a tripartite protein structure comprising the homologous chromosome axes brought into close apposition by polymerization of transverse filaments. This chromosome remodeling is closely coordinated with homologous recombination, which leads to the formation of genetic crossovers (COs) in late prophase I. At the end of prophase I, the SC is disassembled, allowing the CO sites to be visible cytologically as chiasmata as the homologue pairs (bivalents) condense before the first meiotic division.

**Figure 1.**
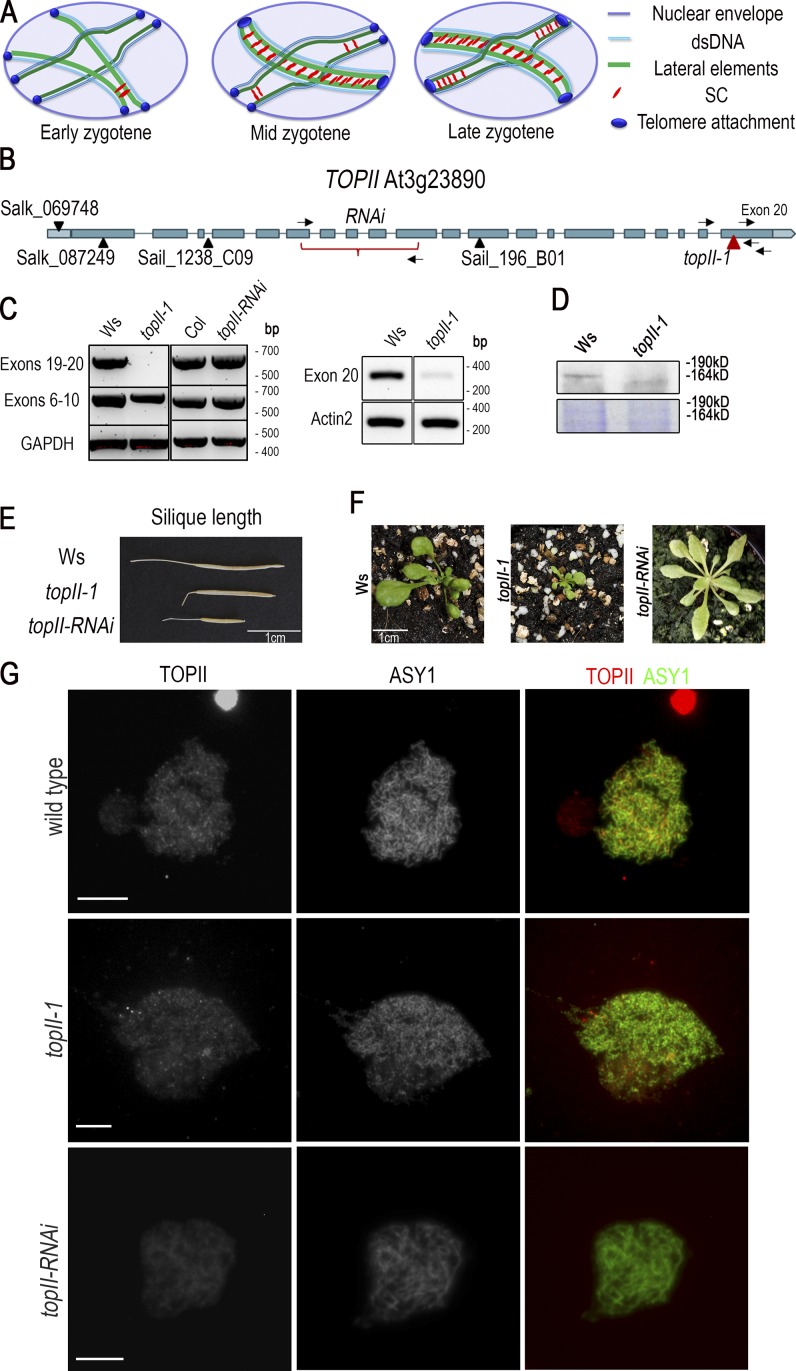
**Molecular and phenotypic analysis of *topII-1* and *topII-RNAi* lines. (A)** Schematic progression of interlock formation from early to late zygotene. In the nucleus, numerous entanglements can impede SC polymerization. When two synapsis initiation sites are formed in between homologous chromosomes with another bivalent or chromosome in between, an interlock arises, and synapsis is delayed in that area. dsDNA, double-stranded DNA. **(B)** *AtTOPII* gene structure and localization of *topII* mutations. Boxes in dark blue indicate exons; lines joining the boxes indicate introns; boxes in light blue indicate 5′ and 3′ UTRs. Arrows represent primers used for RT-PCR. **(C)** Expression of *TOPII* assessed by RT-PCR of buds of *topII-1* and *topII-RNAi* and their respective WT backgrounds. Exons 19–20 amplify the region before and after the T-DNA insertion in *topII-1*. Exons 6–10 correspond with the region complementary for the RNAi construct. Exon 20 amplifies the coding region immediately after the T-DNA insertion. GAPDH and Actin2 are constitutive controls. **(D)** TOPII localization by Western blotting in floral tissue from WT (Ws) and *topII-1.* Section from ∼150–200 kD. Coomassie staining is shown as a loading control. **(E)** Representative silique length comparison of Ws, *topII-1*, and *topII-RNAi*. **(F)** Representative pictures of 33-d WT (Ws), *topII-1*, and *topII-RNAi* plants. **(G)** Immunolocalization of TOPII (mouse) and ASY1 in prophase I of WT (Ws), *topII-1*, and *topII-RNAi* meiocytes using widefield fluorescence microscopy. Bars, 5 µm.

During installation of the SC in the zygotene stage of prophase I, unrelated chromosomes can become interlocked. Interlocks arise when a bivalent or chromosome becomes entrapped between a different pair of homologues undergoing synapsis at each end (Fig. 1 A). It is essential that interlocks are removed in order to complete pairing and avoid possible restrictions to chromosome segregation. Interlocks were first observed in the flatworm *Dendrocoelum lacteum* (Gelei, 1921). Although interlocks appear rare in species with small chromosomes (Holm et al., 1981; Storlazzi et al., 2010), ultrastructural analysis of meiocytes at zygotene and pachytene in species with larger chromosomes such as some insects, mammals, and plants have shown that they occur at high frequency and can involve multiple chromosomes (von Wettstein et al., 1984; Zickler and Kleckner, 1999; Wang et al., 2009). Nevertheless, their presence in meiocytes at pachytene is rare. For instance, in the silkworm *Bombyx mori*, ∼2–3% of cells at pachytene were found to have interlocks, whereas at zygotene, the figure was 87% (von Wettstein et al., 1984). Interlocks have been classified into two categories: the first, in which a bivalent is entrapped in a loop between two stretches of SC of another bivalent, and the second, where an unsynapsed chromosome is trapped (Gelei, 1921). Complex interlocks involving more than two bivalents also occur (Sax and Anderson, 1934; Wang et al., 2009). If a chromosome or bivalent interlocked between two COs remains unresolved, the link can persist until metaphase I without affecting segregation (Heslop-Harrison and Bennett, 1985), but complex structures can impede correct orientation of centromeres (Yacobi et al., 1982). Bivalents interlocked at metaphase I appear as chains of rings or as a rod bivalent in between a bivalent with two chiasmata.

Suggestions have been made to account for the dramatic reduction in the frequency of interlocks observed at pachytene and metaphase I compared with zygotene. Although it is possible that this is simply due to the failure of zygotene cells with interlocks to progress to pachytene or that progression is delayed, leading to an overestimation of the relative abundance of these cells, substantial cytological evidence supports the active removal of interlocks (Zickler and Kleckner, 1999). Ultrastructural studies in human spermatocytes, lily, and *B. mori* have revealed breaks in the chromosome axes and SC at zygotene that are clearly associated with entanglements. By pachytene, these breaks are no longer observed. Hence, it is proposed that formation of a transient chromosomal break allows resolution of the interlock by passage of the trapped chromosome or bivalent, after which the break is rejoined. It is suggested that this might be accomplished by a type II topoisomerase (TOPII), although this would require first remodeling the meiotic axis to create an environment in which multiple DNA strand–passage events by TOPII would be possible (Holm and Rasmussen, 1980; Zickler and Kleckner, 1999). Consistent with this possibility, immunolocalization studies reveal that TOPII is associated with the chromosome axes during meiotic prophase I in mammals and in budding yeast (Klein et al., 1992; Liang et al., 2015; Guturi et al., 2016). Use of TOPII inhibitors in mouse male and female meiocytes lead to defects in chromosome condensation and segregation at the first meiotic division (Kallio and Lahdetie, 1996; Russell et al., 2004; Gómez et al., 2014). Different *topII* conditional mutants in budding yeast and mammals undergo checkpoint arrest in mitosis and meiosis (Holm et al., 1985; Rose and Holm, 1993; Mundbjerg et al., 2015). However, a direct link between TOPII and interlock removal has thus far not been demonstrated.

Another proposal to explain interlock removal invokes chromosome movement toward the end of the other bivalent combined with de- and repolymerization of the SC and telomere detachment from the nuclear envelope (NE) in order to liberate the trapped chromosome (Rasmussen and Holm, 1980). In support, these authors reported the observation of lily meiocytes at early zygotene, where 14 of the 48 telomeres were not associated with the NE. A variation of this model suggests that instead of detaching homologous telomeres from the NE, simply unpairing them would create a gap through which the trapped chromosome could escape (Kleckner and Weiner, 1993). Evidence for this has been found in maize (Golubovskaya et al., 2002), in *sun1sun2* mutants in *Arabidopsis thaliana* (Varas et al., 2015), and in disruption of the nuclear lamina in *Caenorhabditis elegans* (Link et al., 2018).

In this study, we describe the identification of a viable hypomorphic *topII* mutant of the plant *A. thaliana* coupled with meiosis-specific knockdown of TOPII activity using RNAi. Analysis of these lines using a combination of epifluorescence microscopy and SIM has enabled us to confirm a key role for the protein in interlock resolution. Moreover, further analysis using a mutant defective in a nucleoporin, NUP136, which compromises chromosome movement, reveals that although TOPII localization is required for the removal of a proportion of interlocks, chromosome movement is also necessary. Thus, our study indicates that at least two mechanisms operate to remove interlocks during zygotene stage in order to correctly form the SC between homologous chromosomes.

## Results and discussion

From a set of five *topII* transfer DNA (T-DNA) insertion lines, only one, FLAG_476H07 (herein after *topII-1*), present in the Wassilewskija (Ws) ecotype, could be recovered as a homozygous mutant (Fig. 1 B). Previous attempts to identify homozygous mutants from four other lines were also unsuccessful (Makarevitch and Somers, 2005). The T-DNA in *topII-1* is inserted at position 5,927 bp within the last exon, generating an early stop codon that leads to a deletion of the last 82 amino acids of the protein. RT-PCR expression analysis confirmed that the amino terminal region is expressed at near-WT levels in *topII-1,* whereas expression spanning the T-DNA is not detectable (Fig. 1 C). However, a low level of transcription (2%) of the coding sequence downstream of the T-DNA is detectable in buds (Fig. 1 C; Fig. S1 A shows seedling RT-PCR). RT-PCR analysis using T-DNA left-border primer LB4 and TOPII exon 20 primer indicates that this arises from transcription from within the T-DNA, a phenomenon that is not unusual (Wang, 2008). Western blot analysis of WT floral protein extracts with anti-TOPII antibody detected a product around the size (164 kD) predicted for *A. thaliana* TOPII (Fig. 1 D; Xie and Lam, 1994). This was absent in *topII-1* extracts, where a smeary band of lower molecular weight was detected instead. This suggests that TOPII is truncated and that the mutant protein may be slightly less stable. Studies have suggested that the TOPII C-terminal region is poorly conserved and not essential for the catalytic activity of the protein, although it is implicated in its regulation (Shaiu et al., 1999; Dickey and Osheroff, 2005; Kawano et al., 2016). This probably explains why it was possible to identify viable *topII-1* homozygotes. Vegetative development of *topII-1* plants appeared delayed (Fig. 1 F) but was phenotypically normal by maturity except for a 22% reduction in seeds per silique compared with WT (38.5 ± 0.7 and 49.5 ± 0.9; *n* = 25 per genotype; P = 0.001; Fig. 1 E). Given the apparent lethality of most mutant *topII* alleles, we sought to specifically deplete expression of the gene in meiocytes using RNAi. A *TOPII* RNAi cassette based on exons 6–10 was placed under the control of the meiosis-specific *DMC1* promoter in ppF408, which has previously been used for meiosis-specific gene-knockdown research (Fig. 1 B; Higgins et al., 2005). The selected line, *topII-RNAi,* showed a 92% reduction in fertility relative to WT Col-0 (4 ± 0.47; *n* = 30; P < 0.001) but had sufficient residual fertility to enable it to be maintained (Fig. 1 E). RT-PCR analysis revealed apparently normal expression of *TOPII* in buds (Fig. 1 C), but this was anticipated as meiocytes constitute <1% of anther tissue (Chen et al., 2010). Immunostaining using anti-TOPII showed general staining in the chromatin and some punctate signals in WT and *topII-1* but reduced intensity in *topII-RNAi* meiocytes (Col, 433.34 ± 22.26, *n* = 8; *topII-RNAi*, 291.89 ± 27.57, *n* = 14; P = 0.013; Fig. 1 G). The same pattern was confirmed in *Brassica oleracea* meiocytes (Fig. S1 B) and using an independent antibody (Agrisera) in WT and *topII-1* (Fig. S1 C).

### *topII-1* and *topII-RNAi* exhibit increased interlocks at metaphase I

**Figure 2.**
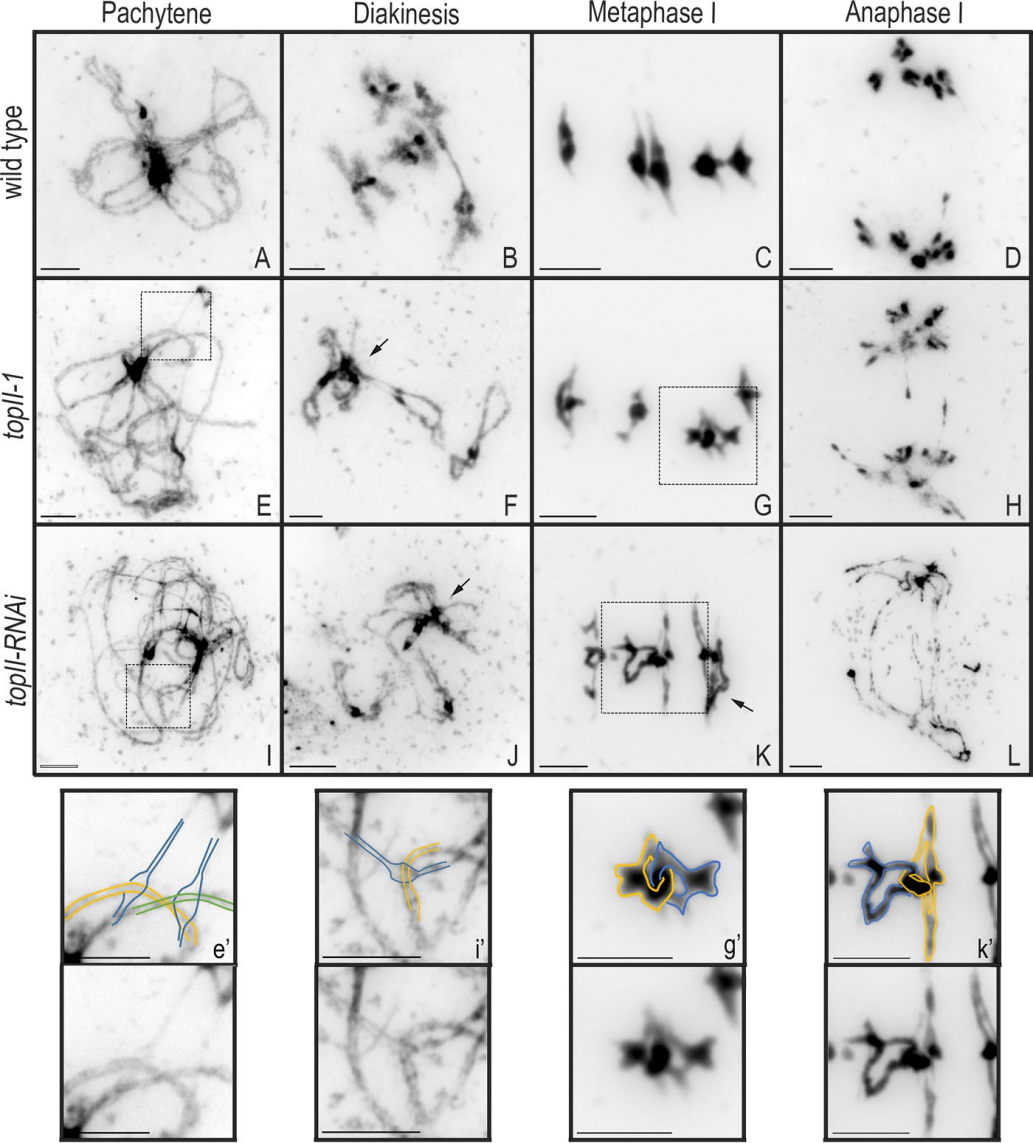
**Chromosome spreads of WT (Ws), *topII-1*, and *topII-RNAi* male meiocytes. (A, E, and I)** Late zygotene/pachytene. **(B, F, and J)** Diakinesis. Arrows indicate excessive chromatin aggregations. **(C, G, and K)** Metaphase I. Arrows indicate interlocks. **(D, H, and L)** Anaphase I. **(e′, i′, g′, and k′)** Amplification of squared regions to illustrate chromosome paths in interlocks in prophase I and metaphase I. Bars, 5 µm.

DAPI-stained chromosome spread preparations from male meiocytes of *topII-1* were examined using epifluorescence microscopy. No obvious differences between *topII-1* and WT (Ws-4) were observed at leptotene. However, we noted apparent interlocks in 57% of *topII-1* late zygotene/early pachytene cells (*n* = 14) compared with 18% (*n* = 17) in WT (Fig. 2, A, E, and e′). It should be noted that it was difficult to reliably distinguish interlocks from chromosome overlaps in a quarter of the cases. Thus, for cells in prophase I, we henceforth use the term entanglement rather than interlock, which we only apply at metaphase I. At diplotene/diakinesis as the SC disassembled, the *topII-1* bivalents were less condensed than WT and often entangled (Fig. 2, B and F). At metaphase I, pairs of interlocked bivalents were present in 20% (*n* = 79) of *topII-1* meiocytes but were absent in WT (Fig. 2, C and G; *n* = 94). A few chromatin bridges were observed in anaphase I nuclei (Fig. 2 H; 1.4 ± 0.3 bridges per cell; *n* = 14). As they involved homologous chromosomes (Fig. S1 D), they are unlikely to arise from an interlock related problem, but they could be a contributory factor in the reduced fertility of *topII-1*.

Further analysis of meiosis revealed that similar numbers of γH2AX foci were present at early zygotene in *topII-1* (187.9 ± 9.3; *n* = 7) and WT (191.5 ± 15.1; *n* = 4; P = 0.927; Fig. S1 E), indicating no effect on double-strand break formation. Similarly, there was no reduction in chiasma frequency relative to WT (8.1 ± 0.2, *n* = 52 vs. 8.2 ± 0.2, *n* = 37; P = 0.574). Meiotic axis length was also unchanged (WT, 291.35 ± 15.28 µm, *n* = 4; *topII-1*, 307.35 ± 12.15, *n* = 7; P = 0.527; Fig. S1 F). At pachytene, SC length was normal in *topII-1* (281.31 ± 7.66 µm) relative to WT (292.94 ± 11.23 µm; *n* = 4 each). Localization of the cohesin SYN1 (Rec8 orthologue) appeared unchanged when analyzed using SIM (Fig. S1 G). Thus, aside from the increase in interlocks, meiosis appears largely normal in *topII-1.*

Examination of *topII-RNAi* meiocytes revealed that at early prophase I, most cells appeared similar to *topII-1* (Fig. 2 I), but in some cases, there was evidence of DNA damage as highly condensed nuclei were observed in slides with zygotene/pachytene stages (12%; *n* = 126), reminiscent of cells undergoing programmed cell death (Fig. S1 H; Kurusu and Kuchitsu, 2017). At diakinesis, 81% of cells had most bivalents aggregated and a more pronounced condensation defect (Fig. 2 J). At metaphase I, interlocks were observed in 88% of the nuclei (*n* = 61; Fig. 2 K). Most cells (54%) had more than two interlocked bivalents (Fig. S1 I), and in nine cases, all five bivalents were involved. Anaphase I and II cells showed high levels of DNA fragmentation, most likely leading to the reduced fertility of *topII-RNAi* plants (Fig. 2 L).

### TOPII is associated with the chromosome axis and accumulates in entangled regions

Immunofluorescence microscopy of chromosome spread preparations of WT meiocytes at zygotene using antibodies that recognize TOPII and chromosome axis protein ASY1 revealed that TOPII mostly localizes as numerous diffuse foci associated with the axis (Fig. 1 G). We observed that in some regions, the foci seemed to accumulate as patches. A TOPII signal was present in *topII-1*, indicating that the mutant protein could associate with chromatin.

**Figure 3.**
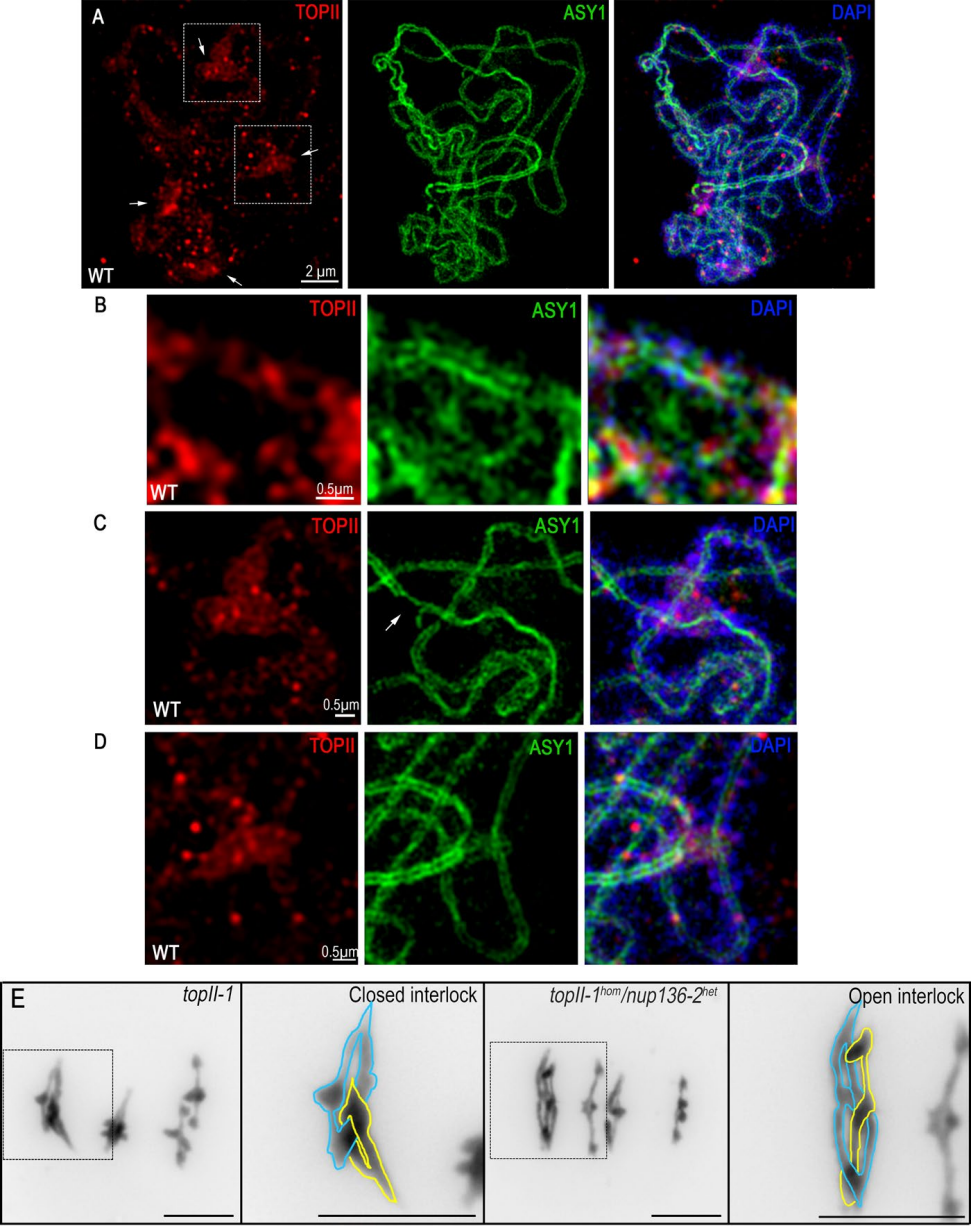
**Immunolocalization of TOPII (red) and ASY1 (green) in WT (Ws) prophase I meiocytes using SIM. (A)** Zygotene stage. Arrows indicate four regions of TOPII accumulation (red) that localize with axis entanglements (green). **(B)** Zoomed region of synapsed axis in Ws. **(C)** Zoomed entangled region with a broken lateral element (green; arrow). **(D)** Zoomed entanglement region with a high accumulation of TOPII. **(E)** Representative examples of “closed” and “open” interlock configurations in *topII-1* and *topII-1^hom^ nup136-2^het^* metaphase I cells and magnified models. Bars, 5 μm.

Further analysis with SIM confirmed the presence of abundant chromatin-associated TOPII foci in WT at zygotene (mean 108.2 ± 10.62; *n* = 5; Fig. 3 A). We also noted that TOPII twisted around the axis in a braid-like manner in a fashion similar to that reported for TOPII distribution in Muntjac mitotic cells (Fig. 3 B; Liang et al., 2015). SIM also suggested that TOPII was enriched in certain regions of the nucleus. Moreover, it appeared (*n* = 23; five cells) that these regions coincided with sites of chromosomal entanglements (Fig. 3, A, C, and D; and Video 1). In some cases, we observed breaks in the chromosome axis in the vicinity of the TOPII-enriched region, reminiscent of those reported in the earlier EM research (Fig. 3 C and Video 2; Holm and Rasmussen, 1980). Although we have not previously observed such breaks, we cannot exclude the possibility that they arose during the spreading procedure. In *topII-1* (Fig. S2, A and B), enrichment of TOPII with entangled regions was less pronounced than in WT (36 entanglements, cells = 4, two of which had a TOPII discrete focus on top, compared with 33 entanglements in WT; Fig. S2 C). Also, the foci were smaller than those in WT (*topII-1*, 25.60 ± 1.79 nm, *n* = 67, vs. 68.90 ± 4.00 nm, *n* = 541; P < 0.001) and less abundant (16.8 ± 6.7 foci per cell; *n* = 4), and the patches of foci observed in WT were absent (Fig. S2). This could suggest that in WT, TOPII is actively recruited to the entangled chromosomes but that the mutation in *topII-1* compromises or reduces this response; however, confirmation requires further study.

### A role for chromosome movement in interlock resolution?

**Figure 4.**
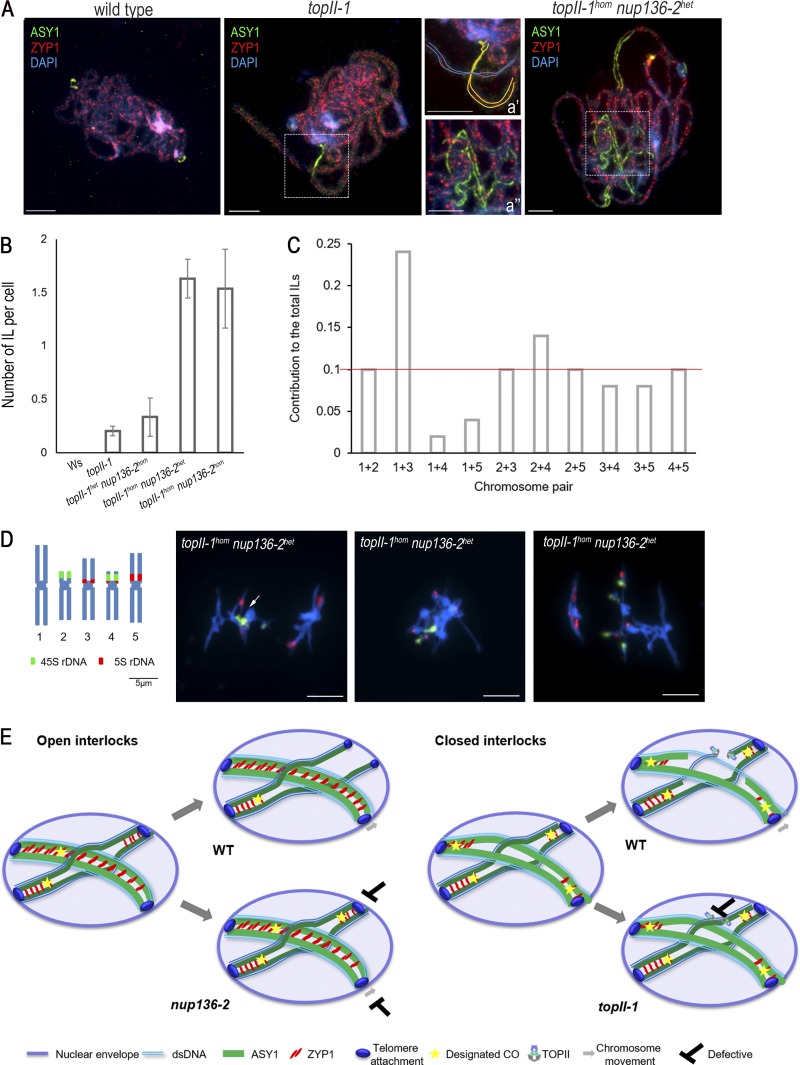
**TOPII and chromosome movement contribute to interlock resolution. (A)** Immunolocalization of SC central element ZYP-1 (red) and lateral element ASY-1 (green) in late zygotene meiocytes of WT, *topII-1*, and *topII-1^hom^ nup136-2^het^*, respectively. **(a′ and a′′)** Zoomed regions to illustrate chromosome paths in interlocks (ILs). **(B)** Mean number of interlocks per cell in WT, *topII-1*, *topII-1^het^ nup136-2^hom^, topII-1^hom^ nup136-2^het^*, and *topII-1 nup136-2* double mutants. *n* > 100, 79, 49, 62, and 13, respectively. **(C)** Frequency of pairs of chromosomes involved in an interlock in *topII-1^hom^ nup136-2^het^* metaphase I cells. *n* = 50 cells. **(D)** Ideogram of FISH of rDNA probes in *A. thaliana* chromosomes and representative metaphase I cells of *topII-1^hom^ nup136-2^het^.* From left to right: two interlocks between bivalents 2 and 4 as well as 1 and 5; all bivalents involved in interlocks and probably other types of connections; and two interlocks between bivalents 2 and 4 as well as 1 and 3. **(E)** Model for interlock-resolution pathways depending on CO designation. Different types of interlocks (“open” vs. “closed”) were found in *topII-1* and *nup136-2*, consistent with interlocks having two resolution pathways. A lack in TOPII would leave unresolved interlocks for which cleavage was essential for their resolution (closed interlocks involving ring bivalents), whereas impaired chromosome movement mutants would produce, mostly, open interlocks. See text for details. Bars, 5 µm. dsDNA, double-stranded DNA.

Inspection of the interlocked chromosomes in *topII-1* revealed that in all 16 cases, they exhibited a “closed” configuration at metaphase I; that is, they involved two or more ring bivalents (those with a chiasmus on either side of the centromere) held together like links in a chain (Fig. 3 E). This configuration likely originated in zygotene, when one of the chromosomes of a bivalent was trapped in the synaptic bubble of another bivalent, and both had CO events at each end (Fig. 4 E). In *topII-RNAi*, 85% (*n* = 59) of the interlocks had a “closed” configuration. As rod bivalents are frequent in *A. thaliana*, interlocks with an “open” configuration would also be expected to arise. In this case, a rod bivalent is entrapped between two flanking chiasmata in another bivalent (Figs. 3 E and 4 D). During zygotene/pachytene, this structure would be maintained by the SC. However, at diplotene/diakinesis, as the SC breaks down and the chromosomes begin to condense, prophase I chromosome movement can potentially release the entrapped rod without a requirement for chromosome breakage, unlike “closed” interlocks. We therefore investigated whether the absence of open interlocks in *topII-1* and *topII-RNAi* might reflect their removal via a separate route dependent on chromosome movement. We examined *nup136-2*, a mutant defective in NUP136 (Tamura et al., 2010), which is required for normal chromosome movement (Varas García, 2014). Although prophase I appeared normal in chromosome spread preparations of *nup136-2* meiocytes (Fig. S3, A–D), examination at metaphase I revealed interlocks in 24.5% (*n* = 49) of the cells. Also, the mutant had a reduced chiasma frequency of 6.0 ± 0.2 (*n* = 40) compared with 8.7 ± 0.2 (*n* = 40) in WT Col-0 (Varas García, 2014), which could presumably reduce the number of interlocks observed at metaphase I. In contrast with *topII-1* and *topII-RNAi,* almost all interlocks involved an open configuration, with only two having a closed configuration (Varas García, 2014). Together with the analysis of *topII-1* and *topII-RNAi*, these observations support a model whereby interlock removal involves both TOPII activity and chromosome movement.

### *topII-1* and *nup136-2* have an additive impact on interlock removal

To gain further support for two mechanisms for interlock removal, we attempted to construct a *topII-1/nup136-2* double mutant. This proved difficult due to the proximity of the loci on chromosome 3 and the impact on viability whereby nearly all progeny died (*n* = 283). Eventually, one surviving double mutant (*topII-1^hom^ nup136-2^hom^*) was identified (Fig. S3, E–H). Although the plant was dwarfed, a few buds were recovered, which allowed the identification of 13 metaphase I cells in chromosome spreads (Fig. S3 C). Examination revealed a chiasma frequency of 6.8 ± 0.4, with an overall mean of 1.5 ± 0.4 interlocks per cell (Table 1). This limited sample suggested that the mutations were additive.

**Table 1.** Proportion of cells with apparent interlocks at late zygotene/pachytene and metaphase I

**Genotype**	**Pachytene**	***n***	**Metaphase I**	***n***
WT	16%	19^a^	0	>100
*topII-1*	44%	9^a^	20%	79
*nup136-2*	28%	18	24% (Varas García, 2014)	49
*topII-1^hom^ nup136-2^het^*	79%	24^a^	69%	62
*topII-1^het^ nup136-2^hom^*	66%	6	25%	12
*topII-1^hom^ nup136-2^hom^*	66%	9	84%	13

a Refers to genotypes analyzed with ASY1 and ZYP1 immunostaining.

Given the poor viability of the double mutant, we investigated the impact of haploinsufficiency in the *topII-1^het^ nup136-2^hom^* and *topII-1^hom^ nup136-2^het^* lines. In the case of the former, no significant vegetative or meiotic differences from the single *nup136-2* mutant were detected (Fig. S3, I–L). The frequency of interlocks at metaphase I remained at 0.33 (*n* = 12; Fig. 4 B). This suggests that in the heterozygote, *TOPII* expression is sufficient to enable plant growth, albeit with the meiotic defects associated with the *nup136-2* homozygote (Fig. S3, A–D). However, the early meiotic phenotype of *topII-1^hom^ nup136-2^het^* was significantly more severe than the *topII-1* homozygote: Late zygotene/early pachytene cells were costained with the SC protein ZYP1 and ASY1 (Fig. 4 A and interpretative cartoon in Fig. 4 a′) to obtain a better quantification of entanglements in WT (16%; *n* = 19), *topII-1* (44%; *n* = 9), and *topII-1^hom^ nup136-2^het^* (79%; *n* = 24), where complete synapsis was often impeded (Fig. 4 a′′), though in fully synapsed cells, SC length was normal (291.52 ± 14.83 µm; *n* = 5). The increased prevalence of interlocks in *topII-1^hom^ nup136-2^het^* was confirmed at metaphase I, where they were found in 69.4% of the cells (*n* = 69). The overall number of interlocks per cell in *topII-1^hom^nup136-2^het^* was 1.63 ± 0.18, which is significantly different from both single mutants (P < 0.001; Fig. 4 B), while maintaining normal levels of chiasmata per cell (8.7 ± 0.1; *n* = 52).

FISH using 45S and 5S ribosomal DNA (rDNA) probes enabled the determination of interlock involvement of each chromosome (Fig. 4, C and D). Unsurprisingly, the two longest chromosomes, 1 and 3, were most frequently involved (in 24% of cases; *n* = 50). However, interlocks between the two short nuclear organizer region–associated chromosomes 2 and 4 were also frequent (14%), probably related to the proximity of these chromosomes in the premeiotic nuclei (Sanchez Moran et al., 2001; Fransz et al., 2002).

We next analyzed the contribution of each type of interlock observed in metaphase I cells of *topII-1^hom^nup136-2^het^.* Unfortunately, interlocks in cells in which most of the bivalents remained entangled proved difficult to reliably classify. Hence, it is possible that as in other mutants with high–interlock frequency factors (Martinez et al., 2001), such as condensation problems, ectopic synapsis, or recombination, may also contribute to formation of these structures. We found one such example in which bivalents 2 and 4 were connected in metaphase I by their 45S region (Fig. 4 D, arrow), probably reflecting ectopic recombination. However, we did not observe terminal connections between chromosomes, indicating nonhomologous end joining activity was not increased. Moreover, mitotic anaphase bridges were not significantly different in *topII-1* (14%; *n* = 194) and *topII-1^hom^nup136-2^het^* (21%; *n* = 207; P = 0.22), suggesting no additional DNA repair defect in this line. As it is likely that closed interlocks are more prone to result in highly entangled chromosomes, their proportion may have been underestimated in the analysis. Nevertheless, of the 50 interlocks that could be classified, 27 had a closed configuration, and 23 were open (Fig. 3 E). Thus, both classes of interlocks remain unresolved when TOPII activity and chromosome movement are both compromised (Fig. 4 E).

In summary, we have provided evidence that chromosome entanglements leading to interlocks during meiosis, first described almost a century ago (Gelei, 1921), are actively removed during meiotic prophase I by at least two distinct pathways working in unison: chromosome movement and a TOPII-regulated pathway. Recent work relates how attachment of chromosomes to the NE avoids entanglements in interphase and meiosis, in line with our findings (Kinney et al., 2018; Link et al., 2018). Questions still remain, particularly in relation to how TOPII activity is regulated to coordinate the different steps in the putative breakage and religation of the chromatids. Moreover, it remains possible that other factors may contribute to interlock removal. For example, directionality toward solving the interlock could be imposed by cohesins (Sen et al., 2016) and distinction between homologous chromosomes by mismatch repair proteins like MLH1. Indeed, a potential role for MLH1 in interlock resolution has previously been proposed based on research in the filamentous fungus *Sordaria macrospora* (Storlazzi et al., 2010).

Our working model proposes that interlock resolution is governed by whether it is a bivalent or a single chromosome that becomes trapped and the relative position of designated COs. For instance, during zygotene/early pachytene, when the telomeres are attached to the nuclear envelop, if a single chromosome of a bivalent with designated COs at each distal end is entrapped between a synapsing bivalent with similarly placed designated COs, then release will require coordinated chromosome and axis breakage followed by break resealing. Failure to do so would result in the presence of closed interlocks at diakinesis/metaphase I. In the case where COs are not designated in all four distal regions, the entrapped chromosome may be released by a combination of chromosome movement and SC depolymerization. In this instance, failure to release the entrapped chromosome would lead to the presence of an open interlock structure. Finally, where a synapsed bivalent is encircled by another synapsing pair of chromosomes, this may be resolved by chromosome movement combined with release (or separation) of the telomeres from the NE (Fig. 4 E).

## Materials and methods

### Plant material and mutants

Plants were grown under controlled temperature and humidity (20°C and 60%) in a mixture of M3 compost, vermiculite, and silvaperl, alternating 16 h light and 8 h darkness (Higgins et al., 2004). For antibiotic resistance selection, sterile seeds were sown in Murashige and Skoog medium (Murashige and Skoog, 1962), 1% sucrose, and phytoagar. Siliques were measured and dissected under a stereomicroscope for fertility assessment. Seeds were provided by Nottingham Arabidopsis Stock Center and the Institute Jean-Pierre Bourgin, and line *nup136-2* (SAIL_796_H02; Tamura et al., 2010) was donated by M. Pradillo (Universidad Complutense, Madrid, Spain). *B. oleracea* plants were variety alboglabra A12DHd. Col or Ws ecotypes were used as a control according to the genetic background of the T-DNA insertion lines used. Lethal insertions in the *TOPII* gene (AT3G23890) included: Sail_1238_C09, Salk_087249, Sail_196_B01, and Salk_069748. The FLAG_476H07 line (*topII-1* allele) displayed a normal segregation assessed by PCR (left genomic primer, 5′-CAGAAGTGGTGAAGCCAAAAG-3′; right genomic primer, 5′-AATCCAAAGAAGACCAGAGGG-3′; and RBTag3, 5′-CTGATACCAGACGTTGCCCGCATAA-3′). Amplification and sequencing of the insertion region in *topII-1* confirmed its position in the last exon and revealed an in-frame early stop codon seven amino acids after the insertion of the T-DNA. TOPII specific region for generating the RNAi construct was amplified by Phusion polymerase using the primers: sense forward, 5′-CTCGAGTGCCTGAATACGAAGAATGGA-3′; sense reverse 5′-GGTACCCCTTTGCCCGTCTTCATTCA-3′; antisense forward, 5′-GGATCCTGCCTGAATACGAAGAATGGA-3′; antisense reverse, 5′-ATCGATCCTTTGCCCGTCTTCATTCA-3′; and pHANNIBAL intron forward, 5′-AGTGATGTGTAAGACGAAGAAGA-3′, and reverse, 5′-AATGATAGATCTTGCGCTTTGTT-3′. DNA extraction buffer (100 mM Tris, pH 9.5, 250 mM KCl, and 10 mM EDTA) was used to obtain genomic DNA. RNeasy minikit and Shredder columns (QIAGEN) were used for RNA extraction. QIAGEN One-Step RT-PCR and Bioline tetrocDNA synthesis kits were used for evaluating mRNA levels of the *AtTOPII* gene with the following primers: TOPIIRTRNAiregion forward, 5′-CAAAATCTCTCGCTTTGGCGGGG-3′; TOPIIRTRNAiregion reverse, 5′-GATTGCCAGGCACATAGCTCG-3′; TOPIIRT19-20 forward, 5′-AGCTGCGAAGGAGGTTGA-3′; TOPIIRT19-20 reverse, 5′-AATCCAAAGAAGACCAGAGGG-3′; TOPIIRT20 forward, 5′-ATCATCTCCGTTCAACAAGAAGAG-3′; TOPIIRT20 reverse, 5′-CATCATCTTCATCGTCTTCAATGTC-3′; GAPDH forward primer (P3), 5′-CTTGAAGGGTGGTGCCAAGAAGG-3′; GAPDH reverse primer (P4), 5′-CCTGTTGTCGCCAACGAAGTCAG-3′; Actin2 forward, 5′-GTGGATATCAGGAAGGATCTG-3′; and Actin 2 reverse, 5′-GTGAACGATTCCTGGACCTGC-3′. As a control for genomic DNA amplification, we used TOPIIRT20-intergenic forward, 5′-CAGTGATGAGCAGGTTGGCTG-3′, and TOPIIRT20-intergenic reverse, 5′-TCGAAGTCCCTCTGCTCTGG-3′, resulting in no specific band amplified. Band intensity was assessed using the ImageJ Plugin Gel Analyzer (National Institutes of Health). Plants were transformed with *pDMC1::TOPIISA* in ppF448 (Higgins et al., 2005) using floral dipping (Clough and Bent, 1998), selected in chloramphenicol, and genotyped by PCR. 25 T1 plants were examined phenotypically. Vegetative growth appeared normal but was accompanied by various levels of reduced fertility, with silique lengths ranging from 0.2 cm to a WT length of 1.4 cm. Although lines with reduced fertility showed similar phenotypes, a representative line (number 24) referred to as *topII-RNAi* was selected for further analysis.

### Protein extraction and immunoblotting

Fresh inflorescences from Ws and *topII-1* were ground in liquid nitrogen, and proteins were extracted by resuspending in 1× SDS-PAGE final sample buffer (62.5 mM Tris-HCl, pH 6.8, 5% [vol/vol] glycerol, 2.5% [vol/vol] β-mercaptoethanol, 2% [wt/vol] SDS, and 0.001% [wt/vol] bromophenol blue) and boiling for 10 min. After centrifuging for 2 min at 15,000 *g* to remove debris, samples were subjected to SDS-PAGE electrophoresis and electroblotted onto nitrocellulose membrane (GE Healthcare). Western blot analysis was performed using α-TOPII (rabbit) at 1:1,000 (Agrisera) followed by α-rabbit–HRP conjugate at 1:10,000 (Sigma-Aldrich). Blots were imaged using enhanced chemiluminescence and film (GE Healthcare).

### Cytogenetic techniques

*A. thaliana* inflorescences were conserved in 3:1 fixative and stored at 4°C for chromosome preparations by spreading technique (Fransz et al., 1998; modified as in Martinez-Garcia and Pradillo, 2017). After cell wall digestion, a single bud was fixed in 60% acetic acid at 45°C and stirred for 1 min. Refixation at 3:1 was followed by drying at RT. Slides were stained with DAPI and mounted in Vectashield. The same procedure was used for FISH, followed by DNA denaturation and hybridization protocol (Sanchez Moran et al., 2001) with the probes 45S rDNA (from plasmid pTa71; Gerlach and Bedbrook, 1979) and 5S rDNA (from plasmid pCT4.2).

*A. thaliana* buds were collected and classified by size and meiotic stage (by lactopropionic orcein staining) in preparation for the immunostaining procedure. Following the protocol of Armstrong et al. (2009), buds were digested on a slide at 37°C (0.4% cytohelicase and 1% polyvinylpyrrolidone) to dissect anthers more easily. Another digestion was followed by mashing the anthers and lysing cells by 1% Lipsol (SciLabware). Cell fixation was performed by incubating with 4% paraformaldehyde until dry. Alternatively, fixed cells were immunostained for the localization of meiotic axis proteins as described by Chelysheva et al. (2013). After washing with ethanol and 1× PBS, slides were boiled in citrate buffer, pH 6. In both cases, slides were incubated with primary antibodies at 4°C for at least 24 h: α-ASY1 (rat or rabbit) at 1:500 (Armstrong et al., 2002), α-ZYP1 (rat or rabbit) at 1:500 (Higgins et al., 2005), α-TOPII (mouse) at 1:100 (Santa Cruz Biotechnology, Inc.), α-TOPII (rabbit) at 1:200 (Agrisera), α-SYN1 (rabbit) at 1:500 (Tiang, 2010), and α-γH2AX at 1:600 (Charbonnel et al., 2011). Slides were washed and incubated with secondary antibodies (α-rat TexasRed, 1:200; α-rat FITC, 1:50; α-rabbitCy3, 1:100; α-rabbit FITC, 1:50; and α-mouse TRITC, 1:200) for 1 h at 37°C and costained with DAPI in Vectashield (Vector Labs). For the structured illumination microscopy (SIM), the same slide preparations were used and incubated with secondary antibodies Alexa Fluor 488 and Alexa Fluor 568 (1:200).

### Image acquisition and processing

For image acquisition, a fluorescent microscope (Nikon i90) equipped with a Nikon DS-Qi1Mc digital camera at RT and NIS Elements software (Nikon) were used. Flat cells on spreading slides were acquired in a Plan Apochromat VC 100× 1.40 NA oil ∞/0.17 differential interference contrast N2 objective by Multichannel acquisition, whereas 3D cells were captured with the Z series acquisition tool using 0.1-µm steps per Z stack. Overexposure was avoided, and exposure time was maintained constant between slides of the same batch for intensity and detailed comparisons. Images were not deconvoluted in any way when using the epifluorescent Nikon microscope except for γH2AX images (MexicanHat). Brightness and contrast were adjusted slightly to remove background but respecting the different intensities in the cell. Adjustments were applied uniformly in mutant and control images. 3D images were processed by maximum-intensity projection for figure presentation.

To analyze the ultrastructure of immunosignals and chromatin beyond the classical Abbe/Raleigh limit at a lateral resolution of ∼120 nm (superresolution; achieved with a 488-nm laser), spatial SIM (3D-SIM) was applied using a 63× 1.4 NA oil Plan Apochromat objective of an Elyra PS.1 microscope system at 30°C and using ZEN Black software (ZEISS). Images were captured separately for each fluorochrome using the 561-, 488-, and 405-nm laser lines for excitation and appropriate emission filters (Weisshart et al., 2016). Maximum-intensity projections of whole meiocytes were calculated with ZEN software. Enlarged sections were presented as single slices to indicate the subnuclear chromatin and protein structures at the superresolution level. 3D rendering based on SIM image stacks was done using the Imaris 8.0 (Bitplane) software.

Pixel intensity (in AU) was measured in total nucleus area in all Z slices per cell with ImageJ. Average intensity per cell was used to compare TOPII signal in WT and *topII-RNAi* samples. The number of TOPII foci per nucleus and their area were measured by ImageJ processing: threshold was adjusted to obtain a binary image without background, watershed was applied in order to individualize the foci, and the Analyze Particles tool gave the results table and the final image with the particles.

### Statistical analysis

Average figures were followed by their standard errors as a deviation measurement. Statistical analysis to compare means was Mann-Whitney U Test performed using SPSS.

### Online supplemental material

Fig. S1 shows RT-PCR gels of seedling material (A), anti-TOPII staining in *B. oleracea* (B), anti-TOPII (Agrisera) staining (C), *topII-1* anaphase I cell with FISH (D), γH2AX localization (E), antiZYP1 staining (F), SYN1 and ASY1 localization by SIM (G), *topIIRNAi* cells examples of damage (H), and interlocks (I). Fig. S2 presents SIM images of TOPII and ASY1 in *topII-1*. Fig. S3 shows chromatin spreading images of *nup136-2, topII-1^hom^ nup136-2^hom^*, and *topII-1^het^ nup136-2^hom^*.

## Supplementary Material

Supplemental Materials (PDF)

Video 1

Video 2
