# Supplementary material for: TOPII and chromosome movement help remove interlocks between entangled chromosomes during meiosis
Source: J Cell Biol. 2018 Dec 3;217(12):4070–9. doi: 10.1083/jcb.201803019 (PMC6279386; doi:10.1083/jcb.201803019)
Supplement: Supplemental Materials (PDF) [file JCB_201803019_sm.pdf]

Supplemental material

Martinez-Garcia et al., https://doi.org/10.1083/jcb.201803019

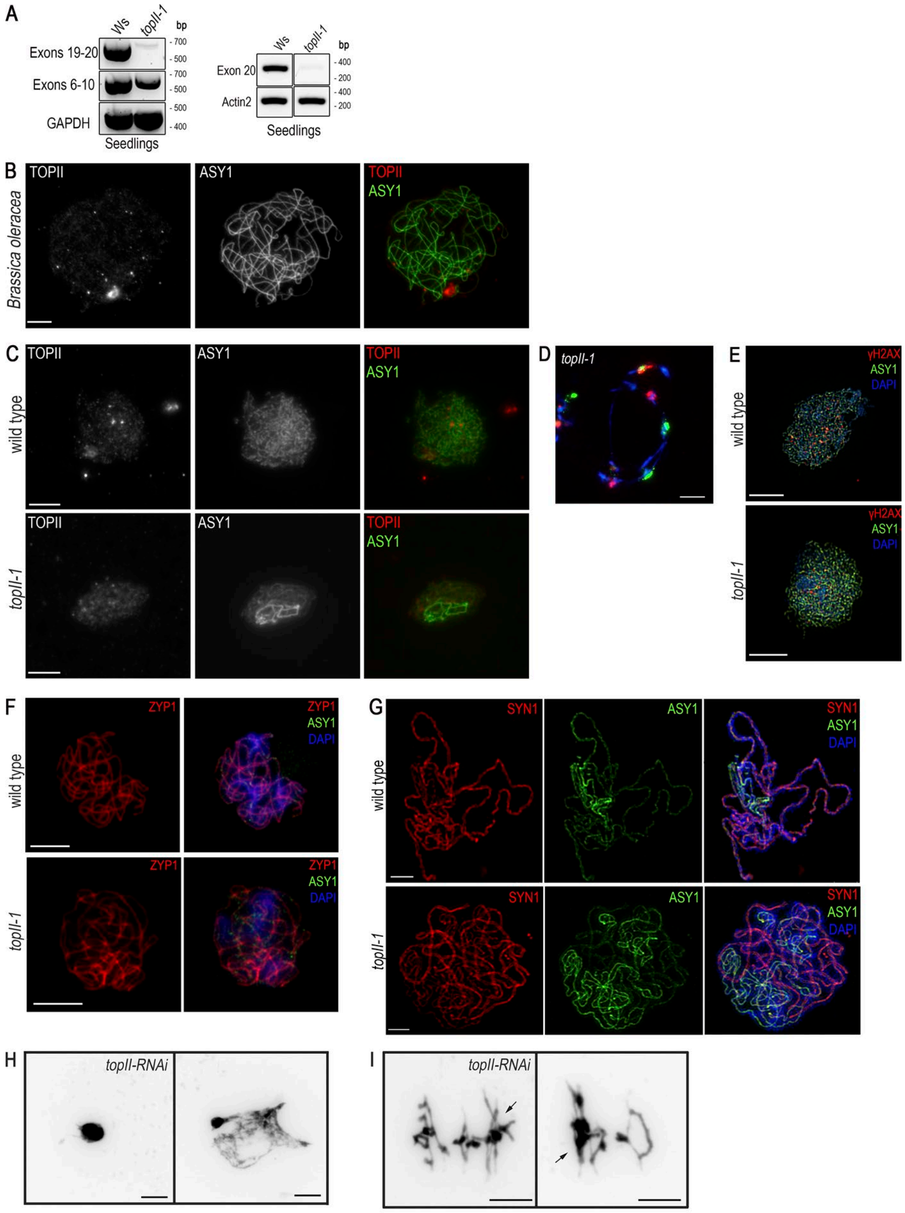

Figure S1. **Cytogenetic analysis of *topII-1* and *topII-RNAi* lines. (A)** Expression of *TOPII* assessed by RT-PCR of seedlings of *topII-1* and WT. **(B)** Immunolocalization of TOPII (mouse) and ASY1 in prophase I cells of *B. oleracea*. **(C)** Immunolocalization of TOPII (rabbit; Agrisera) in WT (Ws) and *topII-1* meiocytes. **(D)** Anaphase I cell of *topII-1* with FISH probes. Bivalents 2 and 4 reveal chromatin bridges between homologues. **(E)** Immunolocalization in early prophase I cells of γH2AX and ASY1 in WT and *topII-1*. **(F)** Immunolocalization in fully synapsed cells of ZYP1 and ASY1 in WT and *topII-1*. **(G)** Immunolocalization in zygotene cells of SYN1 and ASY1 in WT and *topII-1* by SIM. **(H)** Representative chromosome spread images of *topII-RNAi* PMCs with highly condensed nuclei during zygotene and diplotene. **(I)** Representative chromatin spreading images of *topII-RNAi* metaphase I cells with interlocks (arrows). Bars: 5 µm (B–E and G–I); 2 µm (F).

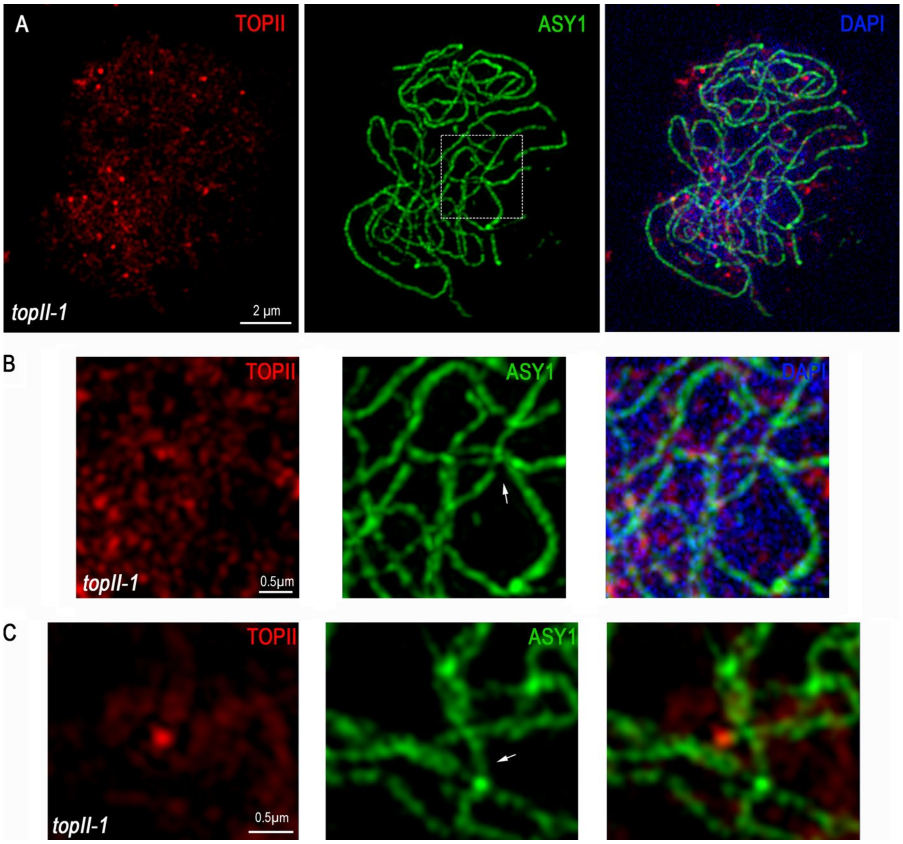

Figure S2. **Immunolocalization of TOPII (red) and ASY1 (green) in prophase I of *topII-1* meiocytes using SIM. (A)** Late zygotene stage. **(B)** Zoomed region with axis entanglements (arrow) without TOPII (red) accumulation. **(C)** Zoom in an interlock (arrow) with a dot of TOPII signal.

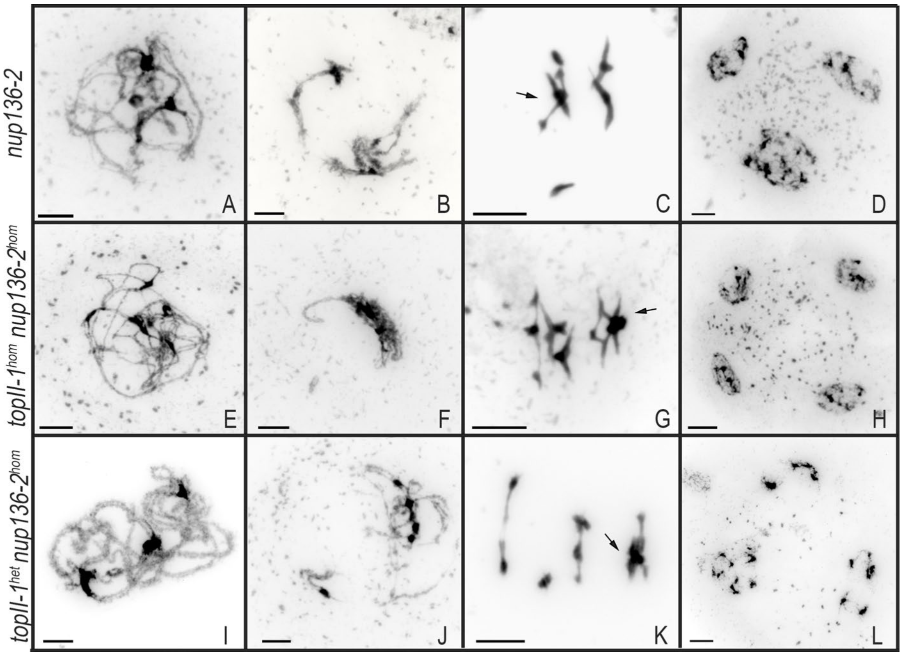

Figure S3. **Representative chromatin spreading images of*nup136-2*, *topII-1*^{hom} *nup136-2*^{hom}, and *topII-1*^{het} *nup136-2*^{hom} PMCs. (A–D)** *nup136-2* PMCs. **(E–H)** *topII-1*^{hom} *nup136-2*^{hom} PMCs. **(I–L)** *topII-1*^{het} *nup136-2*^{hom} PMCs. **(A, E, and I)** Pachytene-stage cells. **(B, F, and J)** Diakinesis stage. **(C, G, and K)** Metaphase I cells with interlocks (arrows). **(D, H, and L)** Tetrad stages.

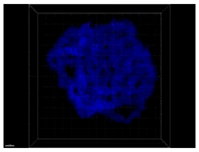

Video 1. **3D rendering of a SIM image stack from a Ws late zygotene cell with TOPII (red) and ASY1 (green) immunolocalization.** Chromatin (blue) was counterstained with DAPI. Bar, 1 µm. Frame rate, 24 frames per second.

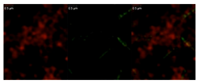

Video 2. **3D-SIM image stack of a Ws meiocyte with an axis break (ASY1 in green) and TOPII accumulation (red).** Chromatin (blue) was counterstained with DAPI. Frame rate, three frames per second.
